# Detection of Nitro-Based and Peroxide-Based Explosives by Fast Polarity-Switchable Ion Mobility Spectrometer with Ion Focusing in Vicinity of Faraday Detector

**DOI:** 10.1038/srep10659

**Published:** 2015-05-29

**Authors:** Qinghua Zhou, Liying Peng, Dandan Jiang, Xin Wang, Haiyan Wang, Haiyang Li

**Affiliations:** 1 Key Laboratory of Separation Science for Analytical Chemistry, Dalian Institute of Chemical Physics, Chinese Academy of Sciences, Dalian, Liaoning, 116023, People’s Republic of China; 2 Jiangsu Province Institute of Quality and Safety Engineering, Nanjing, Jiangsu, 210046, People’s Republic of China; 3 University of Chinese Academy of Sciences, Beijing, 100049, People’s Republic of China

## Abstract

Ion mobility spectrometer (IMS) has been widely deployed for on-site detection of explosives. The common nitro-based explosives are usually detected by negative IMS while the emerging peroxide-based explosives are better detected by positive IMS. In this study, a fast polarity-switchable IMS was constructed to detect these two explosive species in a single measurement. As the large traditional Faraday detector would cause a trailing reactant ion peak (RIP), a Faraday detector with ion focusing in vicinity was developed by reducing the detector radius to 3.3 mm and increasing the voltage difference between aperture grid and its front guard ring to 591 V, which could remove trailing peaks from RIP without loss of signal intensity. This fast polarity-switchable IMS with ion focusing in vicinity of Faraday detector was employed to detect a mixture of 10 ng 2,4,6-trinitrotoluene (TNT) and 50 ng hexamethylene triperoxide diamine (HMTD) by polarity-switching, and the result suggested that [TNT-H]^−^ and [HMTD+H]^+^ could be detected in a single measurement. Furthermore, the removal of trailing peaks from RIP by the Faraday detector with ion focusing in vicinity also promised the accurate identification of KClO_4_, KNO_3_ and S in common inorganic explosives, whose product ion peaks were fairly adjacent to RIP.

Ion mobility spectrometer (IMS) is a well-known tool for gas-phase ion separation based on the differences of compounds in reduced mass, charge, and collisional cross section. Much of its popularity is derived from its successful application in the detection of conventional explosives including 2,4,6-trinitrotoluene (TNT), cyclo-1,3,5-trimethylene-2,4,6-trinitramine (RDX), pentaerythritol tetranitrate (PETN), ammonium nitrate fuel oil (ANFO) and so on^1^,^2^,^3^,^4^,^5^,^6^, which are usually detected by negative ion molecules. In recent years, the peroxide-based explosives, such as triacetone triperoxide (TATP) and hexamethylene triperoxide diamine (HMTD), have been implicated in the terrorist activities due to their easy preparation^7^,^8^. These explosives were better detected by positive ion mode IMS^9^,^10^,^11^. Therefore, to quickly and fully identify different explosive species, a fast polarity-switchable IMS is highly desirable.

The most crucial part of an IMS is its drift tube, which is normally made of a stack of metal guard rings separated by insulated rings^12^,^13^,^14^,^15^,^16^. At present, two commonly used drift tubes are constructed from thick^13^,^14^,^15^ and thin^16^,^17^ metal guard rings, respectively. With the use of these two types of drift tubes, Soppart *et al.* observed that the drift tube with thin metal guard rings possessed more than twice the signal amplitude at a highly increased resolution^12^. Whereas in our polarity-switching test, due to the effect of accumulated charges, a much longer time was needed for the drift tube with thin metal guard rings to recover its IMS signal after polarity-switching (see supplementary information Fig. S1), which indicates that drift tube with thick metal guard rings might be more suitable for constructing a polarity-switchable IMS. However, Liu *et al.* suggested that the homogeneity of electric field in the drift tube was decreased when the thickness of metal guard rings was increased^18^; the inhomogeneity in the electric field could cause divergence of drift velocity for the same kind of ions^12^, which would probably broaden the peak widths and lead to lower resolving power for the IMS.

To improve the resolving power of an IMS, the radial distribution of ions in the drift tube is of valuable information, as it essentially determines the IMS spectrum. Up to date, several works have been carried out to investigate the radial distribution of ions in the drift tube with different ion sources^19^,^20^,^21^,^22^,^23^,^24^. Among these, Eiceman and Davila *et al.* experimentally measured the radial ion density profiles in the ^63^Ni ionization IMS using a charge accumulation IonCCD detector with imaging capability of 2126 pixels at each 21 μm width, suggesting that the ion density was highest at the center of detector while declined radially^19^,^21^. However, this result was in contradiction with the work by Karpas *et al.*^24^ in which the ion density at the center of detector was lower than its outer parts. Tabrizchi *et al.*^23^ and Kwasnik *et al.*^20^ measured the radial ion density profiles in the drift tube using corona discharge ion source, and the ion density profiles were qualitatively similar to the observation of drift tube using electrospray ionization ion source by Hill *et al.*^22^. In practice, Wu *et al.* achieved an improvement of IMS resolving power by decreasing the internal diameter of aperture grid^25^; but reducing the size of a Faraday detector could cause a drop of IMS signal synchronously^12^,^13^. In addition, the resolving power and peak shape for IMS are significantly depended on the construction of the aperture grid as well as the voltage applied^26^,^27^.

In this study, a fast polarity-switchable IMS was built with thick metal guard rings. The ion mobility spectra and the ion density profiles were carefully studied using five concentric Faraday detectors. Based on the ion density profiles with the voltage difference between aperture grid and its front guard ring (ΔV), a Faraday detector with ion focusing in vicinity was developed to boost the IMS signal while removing the tailing peaks from the reactant ion peak (RIP). Finally, the analytical performances of this IMS apparatus for explosives detection were demonstrated by the detection of a mixture of TNT and HMTD via fast polarity-switching between negative and positive ion modes in a single measurement. In addition, the detection of KClO_4_, KNO_3_ and S in common inorganic explosives, such as black powder was achieved.

## Methods

**Figure 1:**
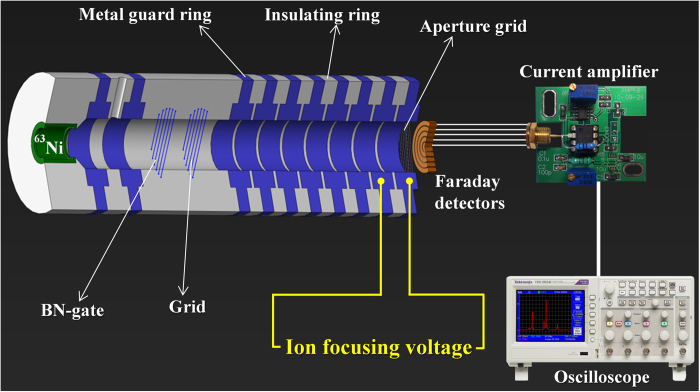
Schematic drawing of ion mobility spectrometer (IMS) constructed with thick metal guard rings.

An IMS apparatus with BN-grid structure^28^ was built using the stack design with thick stainless steel guard rings (18 mm i.d., 8 mm thickness) and Teflon insulating rings (18 mm i.d., 1 mm thickness), schematically shown in Fig. 1. A ^63^Ni foil was used as the ion source in a cylinder (10 mm length, 10 mm diameter). The drift region was 79 mm in length, and a voltage difference of 197 V was applied between the two adjacent guard rings. The aperture grid was made of stainless steel screen (0.2 mm thickness, 40 mesh), 1 mm in front of the Faraday detector. It should be mentioned that the voltage difference between the aperture grid and its front guard ring, ΔV, could be adjusted from 197 to 1379 V, while the voltage difference between the aperture grid and Faraday detector was kept constant at 355 V. The gating voltage difference applied on the BN gate was 220 V, and the gating pulse width was 300 μs. Additionally, a different IMS apparatus was built to merely detect the mixture of TNT and HMTD via fast polarity-switching between the negative and positive ion modes, as a comparative test; this apparatus was similar to that in Fig. 1, except the following parameters: the stack of thin stainless steel guard rings (18 mm i.d., 1 mm thickness) with Teflon insulating rings (18 mm i.d., 6 mm thickness), and the drift region of 67.5 mm in length. When the polarity-switching was carried out, a recovery time of 2 s and 20 s was required for the IMS with the thick and thin metal guard rings, respectively (see supplementary information Fig. S1).

**Figure 2:**
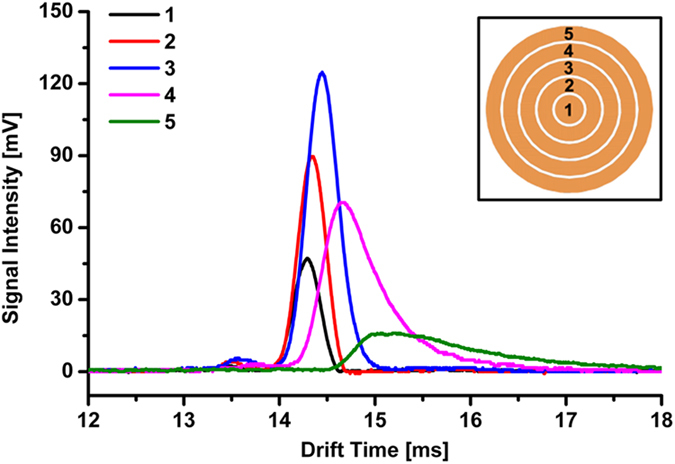
Ion mobility spectra of reactant ion peak (RIP) obtained by five different concentric detectors, the voltage difference between aperture grid and its front guard ring (ΔV) was 197 V.

To investigate the radial distribution of ions in the drift tube with thick metal guard rings at 20 ^o^C, we divided a traditional Faraday detector (radius of 8.7 mm) into five detectors, including a center plate (No.1, radius of 1.5 mm) and four concentric rings (No.2~5, ring width of 1.5 mm each), which were mounted on an insulated support and separated from each other by a space of 0.3 mm, schematically shown as the inset in Fig. 2. The ion currents from the detectors were amplified by the same preamplifier and were acquired by an oscilloscope (Tektronix TDS 2024C). When a specific Faraday detector was connected to the preamplifier for measurement, the other ones were connected to the ground.

Clean air, filtrated by silica gel, activated carbon and 13X molecular sieve traps, was used for the carrier and drift gases, with a flow rate of 300 and 500 mL min^−1^, respectively. The moisture level of purified air was kept below 1 ppm.

50 μg μL^−1^ KClO_4_, 50 μg μL^−1^ KNO_3_, and 0.05 μg μL^−1^ S standard stock solutions were prepared by weighing and dissolving their pure samples in the distilled water or acetone. 1 μg μL^−1^ TNT standard stock solution was purchased from AccuStandard, Inc. (New Haven, CT). HMTD was synthesized in our laboratory by referring to the previous work^29^, and a 1 μg μL^−1^ stock solution was prepared by weighing and dissolving of HMTD in acetone. Test samples were obtained by successive dilution of their standard stock solutions with methanol or acetone, and were introduced into the IMS apparatus at 100 ^o^C by a thermal desorber^1^,^30^ at 180 ^o^C. Firstly, 1 μL of sample solution was deposited on a Teflon-coated fiberglass swab, and then the swab was inserted into the thermal desorber after the solvent was evaporated. The carrier gas passed through the desorber and sent the vapor sample into the IMS apparatus for analysis.

Safety consideration: HMTD is an extremely dangerous substance which may lead to severe explosion, so the synthesis and treatment of HMTD should be only carried out by authorised and highly skilled professional personnel in small quantities, using appropriate protection measures (reinforced gloves and goggles, explosion-proof vessels, etc.).

## Results and Discussion

### Ion radial distribution and peak profiles

Figure 2 illustrates the ion mobility spectra of negative RIPs at five concentric detectors in the drift tube with thick metal guard rings, from which we summarize the peak characteristics in Table 1. Ion density, defined as the division of the area of RIP by the area of detector, is maximal at the central detector, and then gradually declines for the detector away from the central axis of drift tube (the distance was defined as *d*_x_), which agrees to the previous works by Eiceman and Davila^19^,^21^. It is notable that the drift time of RIP shifts to the longer one, and the full width at half maximum (FWHM) of RIP is widened as *d*_x_ is increased. Especially, there are obvious trailing edges appeared for the RIP obtained by detector #4 and #5, which should be attributed to the inhomogeneity of drift electric field near the inwall of drift tube (see supplementary information Fig. S2).

### Traditional Faraday detector

**Figure 3:**
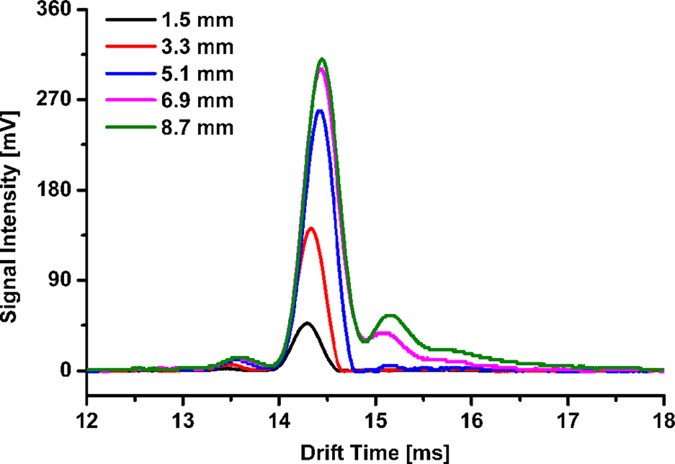
Ion mobility spectra of RIP at the traditional Faraday detectors with radiuses of 1.5, 3.3, 5.1, 6.9, and 8.7 mm, respectively, ΔV = 197 V.

Figure 3 shows the profiles of RIPs at the traditional Faraday detectors with different radiuses of 1.5, 3.3, 5.1, 6.9, and 8.7 mm. The RIP intensity at the Faraday detector with radius of 1.5 mm is 47 mV, which can be improved by factors of 3, 5.5 and 6.4 when the radius of Faraday detector is increased to 3.3, 5.1 and 6.9 mm, respectively; while no prominent improvement of RIP intensity can be observed by further increasing the radius from 6.9 to 8.7 mm. However, when the radius of Faraday detector is larger than 3.3 mm, RIP appears with trailing peaks; from the results in Fig. 2, it can be concluded that these trailing peaks should be produced by the ions of drift trajectories far away from the central axis of drift tube, reaching the Faraday detector with longer drift times.

According to Fig. 3, to obtain the RIP without trailing peaks, it is appropriate to reduce the radius of traditional Faraday detector to 3.3 mm. Nevertheless, comparing to the larger one (radius of 8.7 mm), the RIP intensity at this reduced Faraday detector is decreased by more than 50%, so it is desirable to improve its signal intensity.

### Faraday detector with ion focusing in vicinity

**Figure 4:**
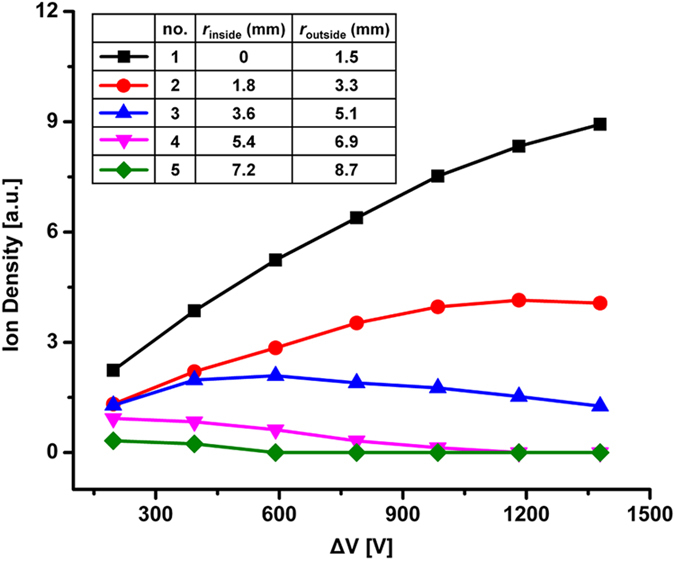
Densities of reactant ion at five different concentric detectors with the increase of ΔV from 197 to 1379 V. Inside radius of the detector: *r*_inside_, outside radius of the detector: *r*_outside_.

As shown in Fig. 4, we measured the density of reactant ion at five concentric detectors with increasing ΔV from 197 to 1379 V, where ΔV was the voltage difference between aperture grid and its front guard ring. Interestingly, at detector #1, the ion density increases constantly with ΔV; at detector #2 and #3, the ion density initially increases then decays at 1182 V and 394 V, respectively; at detector #4 and #5, the ion density decays with increasing of ΔV. To understand these phenomena, we simulated the ion trajectories in drift tube while ΔV was increased from 197 to 1379 V using Simion 8.0 (see supplementary information Fig. S3). The results indicate that ion focusing exists near the aperture grid, and its strength is positively correlated to ΔV. Thus, combining the ion focusing to the reduced Faraday detector (radius of 3.3 mm), the intensity of IMS signal can be significantly enhanced.

**Figure 5:**
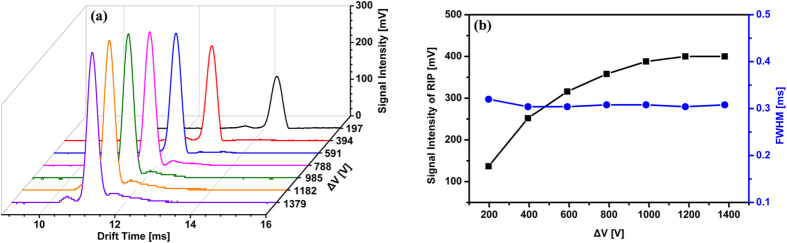
(**a**) Ion mobility spectra of RIP by increasing ΔV from 197 to 1379 V; (**b**) the RIP signal intensity and full width at half maximum (FWHM) versus ΔV using the reduced Faraday detector (radius of 3.3 mm).

As presented in Fig. 5a, we obtained a series of ion mobility spectra using a Faraday detector with radius of 3.3 mm by increasing ΔV from 197 to 1379 V. By plotting the RIP signal intensity and FWHM versus ΔV in Fig. 5b, it can be found that increasing ΔV indeed brings a prominent increase for RIP signal intensity, while the FWHMs of RIP remain at about 0.3 ms. Whereas, when ΔV is higher than 591 V, the ion focusing is so strong that those ions near the inwall of drift tube can be focused to the Faraday detector, leading to the trailing peaks for RIP again, as shown in Fig. 5a.

Therefore, a Faraday detector with ion focusing in vicinity (radius of 3.3 mm, ΔV of 591 V) was selected to produce RIP without trailing peaks, and its intensity was comparable to that obtained by the traditional Faraday detector with radius of 8.7 mm (see supplementary information Fig. S4).

### Detection of TNT and HMTD in a single measurement

To demonstrate the ability of proposed IMS with thick metal guard rings for the detection of nitro-based and peroxide-based explosives in a single measurement, an explosive mixture, consisting of 10 ng TNT (nitro-based explosive, detected by negative ions) and 50 ng HMTD (peroxide-based explosive, detected by positive ions), was chosen to be detected by the polarity-switching method. 1 μL of this explosive mixture was firstly deposited on a Teflon-coated fiberglass swab; after the solvent was evaporated, the swab was inserted into the thermal desorber for 15 s and the polarity-switching of IMS was carried out at the 7^th^ s. The mixture was also detected by the IMS with thin metal guard rings for a comparative test.

**Figure 6:**
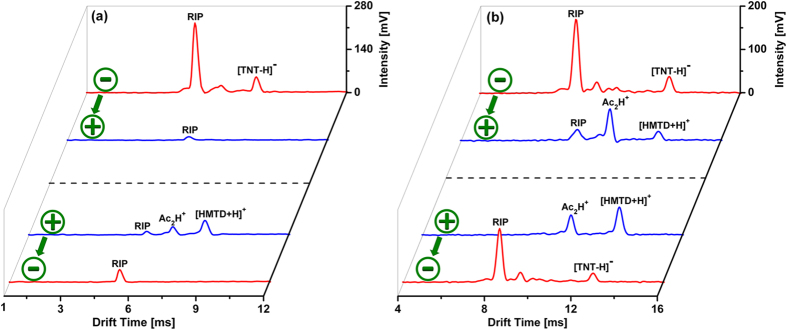
Ion mobility spectra of an explosive mixture including 10 ng TNT and 50 ng HMTD obtained by polarity-switching IMS with (**a**) thin and (**b**) thick metal guard rings. The Ac_2_H^+^ ion peak in the positive spectrum comes from the acetone used to dissolve HMTD.

As shown in Fig. 6a, for the IMS with thin metal guard rings, when the polarity was switched from negative ion mode to positive ion mode, only [TNT-H]^−^ in the negative spectrum could be detected, while no HMTD signal appeared in the positive spectrum. Similarly, when the polarity was switched from positive to negative, only [HMTD+H]^+^ in the positive spectrum could be detected, while no TNT signal appeared in the negative spectrum. Conversely, as shown in Fig. 6b, when the mixture sample of TNT and HMTD was detected using IMS with thick metal guard rings, no matter what polarity-switching sequence it was, [TNT-H]^−^ in the negative spectrum and [HMTD+H]^+^ in the positive spectrum could be both detected, achieving the detection of TNT and HMTD in a single measurement.

### Identification of inorganic explosives

In the last two decades, inorganic explosives have been employed in many terrorist attacks^31^, due to their unrestricted availability and low cost. Using IMS to identify the typical components in inorganic explosives, such as KClO_4_, KNO_3_ and S, it is found that their product ion peaks are fairly closed to the RIP^30^, so a RIP without trailing peaks is required to accomplish their identifications. To demonstrate the capacity of proposed IMS with ion focusing in vicinity of Faraday detector for inorganic explosives detection, 600 ng KClO_4_, 4 ng KNO_3_ and 2 ng S were chosen to be detected in the negative ion mode. To facilitate the evaporation of these compounds during the thermal desorption introduction, 15 μL of 3% H_3_PO_4_ solution was added into 1 mL of their test solutions^30^.

**Figure 7:**
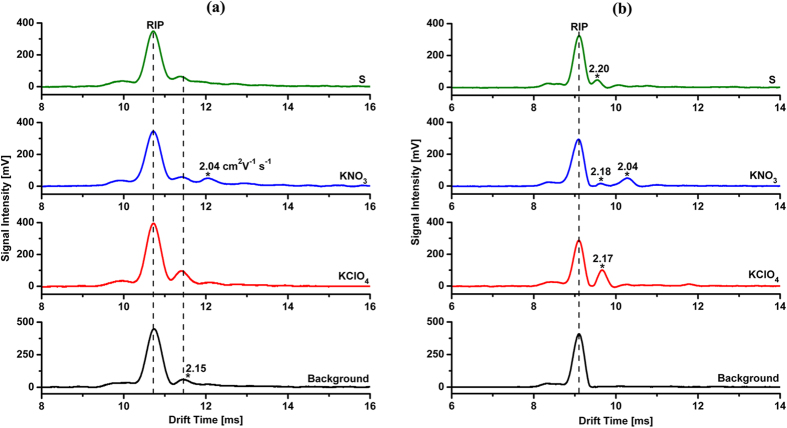
Ion mobility spectra of 600 ng KClO_4_, 4 ng KNO_3_ and 2 ng S using (**a**) large traditional Faraday detector (radius of 8.7 mm) and (**b**) Faraday detector with ion focusing in vicinity. To facilitate the evaporation of these compounds during the thermal desorption introduction, 15 μL of 3% H_3_PO_4_ solution was added into 1 mL of their test solutions.

As a comparison test in Fig. 7a, with the large traditional Faraday detector (radius of 8.7 mm), the RIP is accompanied with a trailing peak (reduced mobility *K*_0_ = 2.15 cm^2^ V^−1^ s^−1^), which are seriously overlapped with the ion peaks of KClO_4_ and S, so only KNO_3_ could be identified by the ion peak with *K*_0_ of 2.04 cm^2^ V^−1^ s^−1^. Conversely, using the Faraday detector with ion focusing in vicinity, the trailing peak of RIP is disappeared, achieving the reliable identification of ion peaks for KClO_4_ (*K*_0_ = 2.17 cm^2^ V^−1^ s^−1^), KNO_3_ (*K*_0_ = 2.18 and 2.04 cm^2^ V^−1^ s^−1^) and S (*K*_0_ = 2.20 cm^2^ V^−1^ s^−1^), as shown in Fig. 7b. What is more, the peak intensities in Fig. 7b are comparable to that in Fig. 7a, suggesting that the use of Faraday detector with ion focusing in vicinity would not cause visible loss of detection sensitivity.

## Conclusions

In this work, we developed a fast polarity-switchable ion mobility spectrometer with ion focusing in vicinity of Faraday detector for explosives detection. By fast polarity-switching, nitro-based explosives such as TNT and peroxide-based explosives such as HMTD could be detected in a single measurement, which broadened the detectable explosive species. Furthermore, the use of Faraday detector with ion focusing in vicinity could remove trailing peaks from RIP, providing a simple method to accurately identify the typical components in inorganic explosives whose product ion peaks were fairly adjacent to RIP, without loss of detection sensitivity.

## Additional Information

**How to cite this article**: Zhou, Q. *et al.* Detection of Nitro-Based and Peroxide-Based Explosives by Fast Polarity-Switchable Ion Mobility Spectrometer with Ion Focusing in Vicinity of Faraday Detector. *Sci. Rep.* **5**, 10659; doi: 10.1038/srep10659 (2015).

## Supplementary Material

Supporting Information

## Figures and Tables

**Table 1:** Characteristics of RIPs at five different concentric detectors.

Detector	Drift Time	Height	FWHM	Ion density
number	/ms	/mV	/ms	
1	14.29	47	0.32	2.24
2	14.33	90	0.34	1.32
3	14.45	125	0.39	1.26
4	14.64	70	0.66	1.02
5	15.02	16	1.44	0.40
