# Supplementary material for: Detection of Nitro-Based and Peroxide-Based Explosives by Fast Polarity-Switchable Ion Mobility Spectrometer with Ion Focusing in Vicinity of Faraday Detector
Source: Sci Rep. 2015 May 29;5:10659. doi: 10.1038/srep10659 (PMC4448110; doi:10.1038/srep10659)
Supplement: Supporting Information [file srep10659-s1.pdf]

Supplementary Information (S-1)

Detection of Nitro-Based and Peroxide-Based Explosives by Fast Polarity-Switchable Ion Mobility Spectrometer with Ion Focusing in Vicinity of Faraday Detector

Qinghua Zhou,[†,§] Liying Peng,[†,§] Dandan Jiang,[†,§] Xin Wang,[†] Haiyan Wang,[‡] and Haiyang Li[†,]*

[†]Key Laboratory of Separation Science for Analytical Chemistry, Dalian Institute of Chemical Physics, Chinese Academy of Sciences, Dalian, Liaoning, 116023, People's Republic of China

[‡]Jiangsu Province Institute of Quality and Safety Engineering, Nanjing, Jiangsu, 210046, People's Republic of China

[§]University of Chinese Academy of Sciences, Beijing, 100049, People's Republic of China

*E-mail: hli@dicp.ac.cn. Fax: +86-411-84379517.

ABSTRACT

This supplementary information provides additional information on the following aspects:

In this study, two ion mobility spectrometer (IMS) apparatuses were constructed with drift tubes using thick metal guard rings (8 mm in thickness) separated by Teflon rings (1 mm in thickness) and using thin metal guard rings (1 mm in thickness) separated by Teflon rings (6 mm in thickness), respectively. Once these two apparatuses were switched from the positive ion mode to the negative one, the reactant ion peak (RIP) intensity was continuously monitored, the time RIP intensity arrived at 50% maximum was defined as the recovery time ($t_{1/2}$). As shown in Fig. S1, $t_{1/2}$ for IMS with thick metal guard rings is shorter than 2 s while that for IMS with thin metal guard rings is about 20 s. Comparing to the drift tube with thick guard rings, the inwall material of the drift tube with thin metal guard rings is most of Teflon; whereas Teflon is well-known to collect charges, thus, more charges will be accumulated on its inwall and a longer recovery time will be needed when the polarity-switching is carried out.

As shown in Fig. S2, the electric field in the drift tube with thick metal guard rings was simulated using Simion 8.0, from which we can see the prominent inhomogeneity of electric field near the inwall of drift tube.

As shown in Fig. S3, we simulated the ion trajectories in drift tube with thick metal guard rings while the voltage difference between the aperture grid and its front guard ring (ΔV) was increased from 197 to 1379 V using Simion 8.0. It is clear that with

increasing ΔV, ions in the drift tube can be focused near the aperture grid, and the focusing strength was positively correlated with ΔV.

As shown in Fig. S4, we detected the ion mobility spectra of RIP using traditional Faraday detector (radius of 8.7 mm) and Faraday detector with ion focusing in vicinity, respectively.

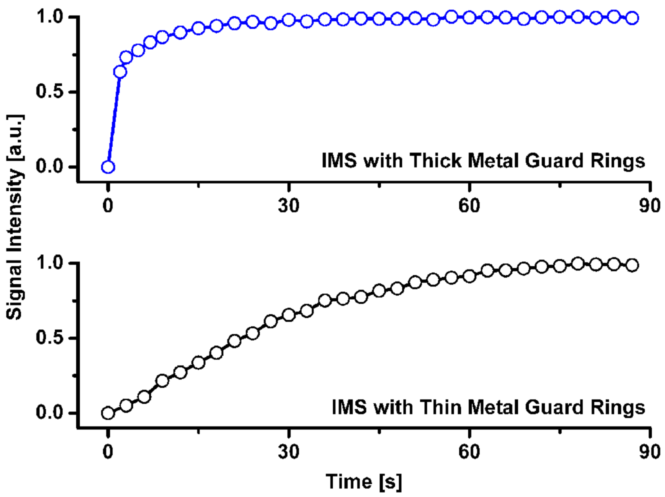

Figure S1. After polarity-switching from positive ion mode to negative one, temporal profile of RIP intensity for IMS with thick and thin metal guard rings, respectively.

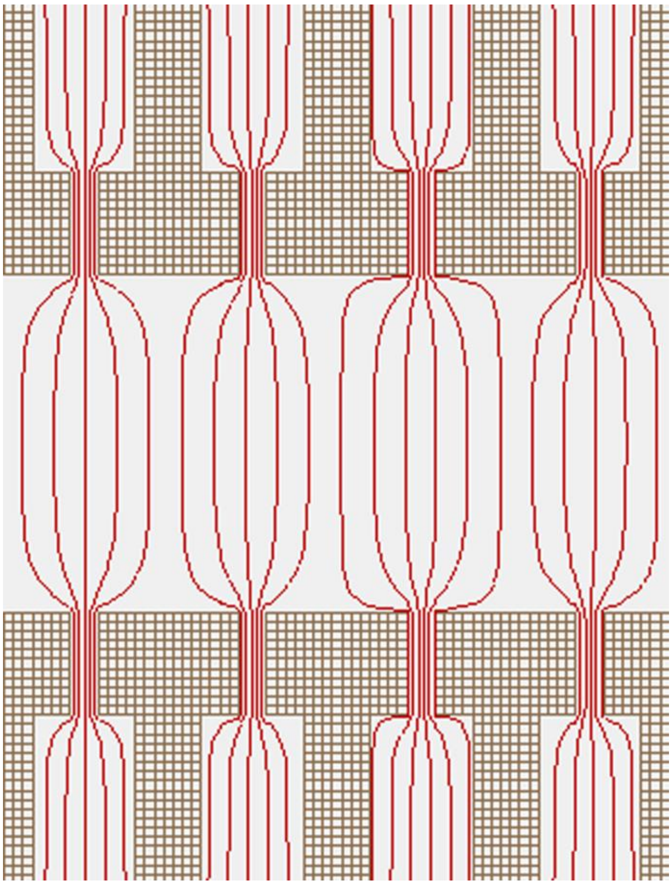

Figure S2. Electric field in the drift tube with thick metal guard rings.

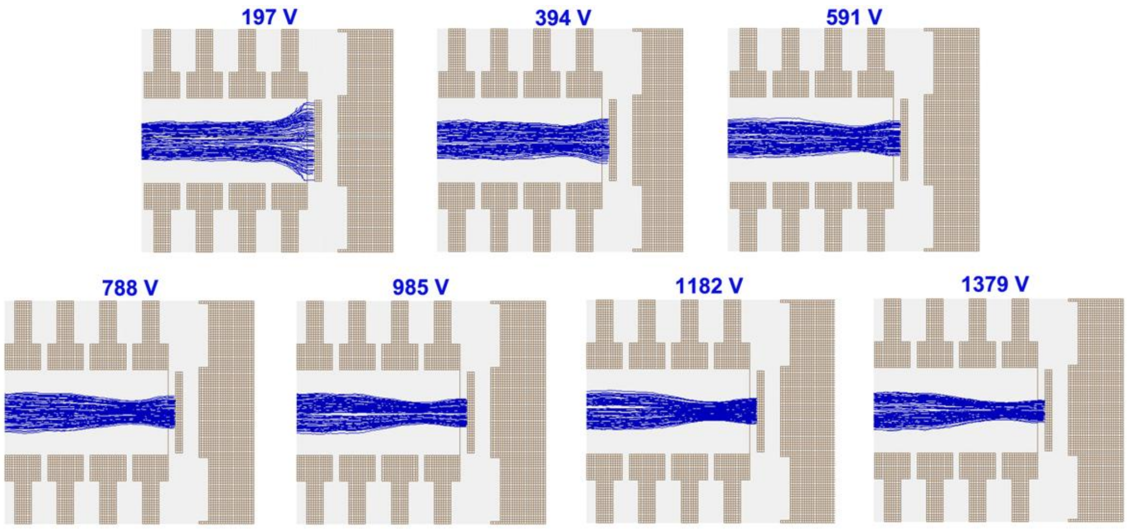

Figure S3. Simulation of ion trajectories in the drift tube with thick metal guard rings by increasing the voltage difference between aperture grid and its front guard ring (ΔV) from 197 to 1379 V using Simion 8.0.

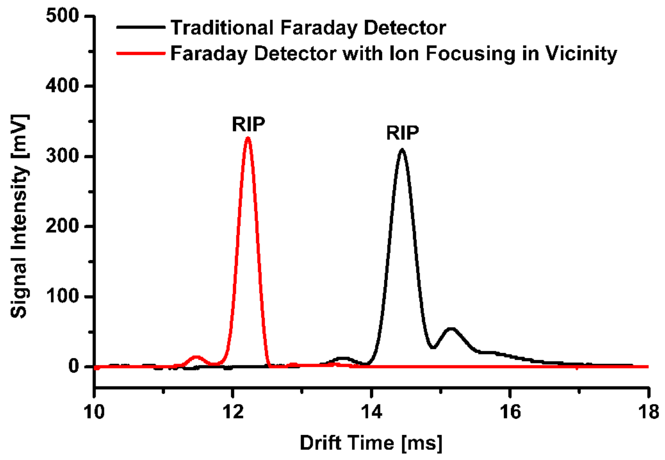

Figure S4. Ion mobility spectra of RIP at the traditional Faraday detector (radius of 8.7 mm) and the Faraday detector with ion focusing in vicinity, respectively.
